# Bioinformatics Analysis of Inflammation Gene Signature in Indicating Cholangiocarcinoma Prognosis

**DOI:** 10.1155/2022/9975838

**Published:** 2022-08-27

**Authors:** Yanting Wang, Shi Chen, Song He

**Affiliations:** ^1^Department of Gastroenterology and Hepatology, The Second Affiliated Hospital of Zhejiang University School of Medicine, Hangzhou, Zhejiang, China; ^2^Department of Oncology, Chengdu Jinniu District People's Hospital, Chengdu, Sichuan, China; ^3^Department of Hepatobiliary Surgery, The Affiliated Hospital of Xiangnan University, Chenzhou, Hunan, China

## Abstract

**Aim:**

We studied inflammatory response-related genes in cholangiocarcinoma by bioinformatics analysis.

**Methods:**

The expression profiles and clinical information of cholangiocarcinoma patients were downloaded from the TCGA cohort and the Gene Expression Omnibus. The greatest absolute shrinking and selecting operator Cox analyses were utilized to build a multigene predictive signature.

**Results:**

An inflammation response-related gene profile was generated using LASSO-Cox regression analysis of Homo sapiens bestrophin 1 (BEST1), Chemokine (C–C motif) ligand 2 (CCL2), and plasminogen activator, urokinase receptor (PLAUR). Individuals in the highest category had a significantly lower overall survival time than those from the low-risk group. A receiver operating curve analysis was used to demonstrate the predictive ability of the predictive gene signature. Through multivariate Cox analysis, the risk score was discovered to be a predictor of overall survival (OS). According to functional assessments, the immunological state and milieu of the two risk areas were significantly different. The expression levels of predictive genes were found to be strongly linked to the sensitivity of cancer cells to antitumor therapy.

**Conclusion:**

A new signature made up of three respective response-relevant genes is found to be a promising indicator of prognosis by influencing the immune condition and tumor microenvironment.

## 1. Introduction

Hepatocellular carcinoma and cholangiocarcinoma are common primary cancers of the liver responsible for an increasing number of cancer-related deaths [1]. Cholangiocarcinoma has become more commonly diagnosed across the world in recent decades [2, 3]. Despite numerous improvements in recent decades to better comprehend the pathology of cholangiocarcinoma, its prognosis remains poor [4, 5]. Surgery is the optimal method for patients with limited, resectable cholangiocarcinoma. Yet, the prognosis for these patients remained poor, with a median overall survival (OS) ranging from 12 to 31 months [6]. With the well-established correlation between inflammation and cancer, the inflammatory role in the onset and progression of cancer has long been the subject of current studies. Inflammation can both promote and prevent cancer growth [7]. Scientists can investigate the association between cancer and inflammatory indicators by assessing parameters that are typically available in the blood [8, 9]. The Glasgow Prognostic Score, which includes C-reactive protein and albumin, exhibits independent predictive significance in cancer patients [10]. A growing number of studies utilize various acute-phase proteins to create comprehensive predictive scores for malignancies based on inflammation. Some inflammatory response-related genes, in addition to serum indicators, were employed to predict the metastatic potential and prognosis of hepatocellular carcinoma [10]. However, the link between inflammatory response-related genes and the prognosis of cholangiocarcinoma remains unstudied.

The use of 3D bioprinting to reconstruct tumor microenvironments could be exploited to develop novel antitumor medications. [11] Inflammatory response genes are associated with tumor microenvironments and antitumor drug sensitivity and thus could be exploited in the 3D bioprinting of new antitumor therapies. In this study, we investigated the predictive significance of inflammatory response-related genes in cholangiocarcinoma and generated an inflammatory response-related gene signature. Our study assessed the relationship between the signature and immunological state along with the microenvironment in cholangiocarcinoma.

## 2. Methods

### 2.1. Data Collection

The TCGA cohort and the Gene Expression Omnibus were used to acquire mRNA expression data and clinical information of individuals with cholangiocarcinoma (GEO). GSE107943 was chosen for further analysis after being screened from the GEO database. The fragments per kilobase of exon model per million mapped fragments (FPKM) format were used to acquire data from the GSE107943 dataset. The TCGA RNA-seq transcriptome data were converted to FPKM values. Data from the TCGA and GSE107943 datasets were transformed to normalized counts.

### 2.2. Inflammatory Response-Related Gene Signature

A sample size of 200 genes associated with the inflammatory response was identified, along with their expression profiles. In the TCGA and GEO cohorts, the differentially expressed genes (DEGs) between tumor and nontumor tissues were identified using the R package “limma” with a fold change greater than 2 and a false discovery rate of 0.05. After Bonferroni correction, univariate Cox analysis was utilized to screen for predictive significance in inflammatory response-related genes. To reduce overfitting, our study used LASSO-penalized Cox regression analysis to build a prognostic model. [12, 13] The “glmnet” R package was used to select and reduce variables using the LASSO technique. Using tenfold cross-validation, the penalty parameter of the prognostic model was defined using the minimum criterion (i.e., the value corresponding to the lowest partial likelihood deviation). The risk ratings of patients were calculated using the expression of each inflammatory reaction gene or its corresponding regression coefficient. Based on their median risk scores, patients were divided into high-risk and low-risk groups. By using the R packages “Rtsne” and “ggplot2,” PCA and t-SNE analysis were used to explore the distribution of distinct groups in terms of gene expression levels in the created model. Survival studies of the OS of high and low-risk groups were performed using the R tool “survminer.” The “survival” R package and the “time ROC” R package were used to perform the time-dependent ROC curve method in order to assess the predictive power of the prognosis signature. Univariate and multivariate Cox analyses were used to explore the signature's independent prognostic relevance.

### 2.3. Immune Status and Tumor Microenvironment Analysis

The “GSVA” R package was used to evaluate the invasion scores of 16 immune cells and the activity of 13 immune-related pathways between both the high-risk and low-risk groups using single-sample gene set enrichment analysis (ssGSEA). The levels of immune and stromal malignant cells in various malignant cells were measured using the immune and stromal scores. To investigate whether there exists a link between the risk rating and other scores, the Spearman correlation was applied. [14] To examine if there was a link between the risk score and the immune infiltration subtype, we utilized a two-way ANOVA analysis. Tumor stem cell characteristics were assessed utilizing information extracted from the transcriptome and epigenetics of tumor samples. [15] The Spearman correlation test was used to investigate the relationship between tumor stemness and the risk score.

### 2.4. Chemotherapy Sensitivity Analysis

The NCI-60 database contains 60 distinct cancer cell lines from 9 different types of tumors, including Cholangiocarcinoma, Bladder Cancer, Colorectal Cancer, Esophageal Cancer, Melanoma, Ovarian Cancer, Pancreatic Cancer, Prostate Cancer, and Small Cell Lung Cancer, and was accessed using the CellMiner interface (https://discover.nci.nih.gov/cellminer). Pearson correlation analysis was used to explore the relationship between prognostic gene expression and drug sensitivity. Correlation analysis was used to examine the efficacy of 263 drugs approved by the FDA or in clinical studies.

### 2.5. Statistical Analysis

To compare DEGs between the tumor and adjacent tissues, the Wilcoxon test was performed. The chi-square test was used to compare different proportions. The ssGSEA scores of immune cells and immunological pathways were compared between high-risk groups using the Mann–Whitney test. The Kaplan–Meier analysis was used to compare the differences in OS across subgroups. Univariate and multivariate Cox analyses were used to screen the different factors for OS. The correlations of the prognostic model risk score and the prognostic gene expression level with stemness score, stromal score, immune score, and drug sensitivity were investigated using Spearman or Pearson correlation analysis. R software (version 4.0.3) was used to create the plots, which included the utility, venn, igraph, ggplot2, pheatmap, ggpubr, corrplot, and survminer. In all statistical outcomes, a two-tailed *P* value less than 0.05 indicated statistical significance.

## 3. Results

### 3.1. Prognostic Inflammation-Related DEGs Identification

**Figure 1:**
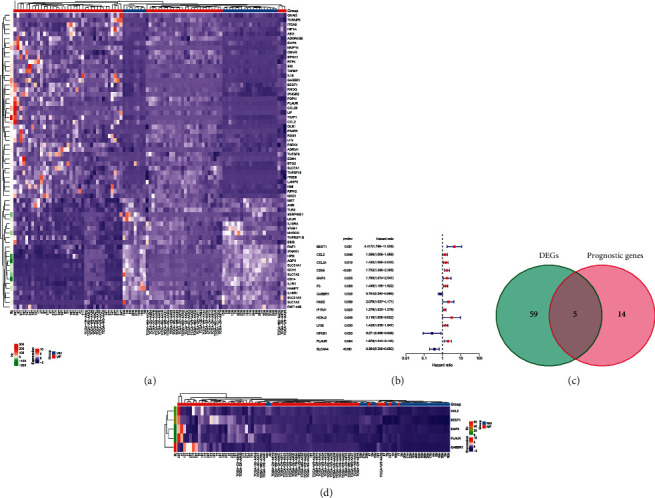
Genes involved in the inflammatory response have been identified as potential candidates. (a) Genes that differ between tumor and nontumor tissues in terms of expression. (b) Forest plots demonstrating 14 genes that linked to patient survival. (c) A Venn diagram was used to determine which genes were differentially expressed and which ones were predictive. (d) Prognostic and differentially expressed genes in tumor and nontumor regions.

Samples of this study consisted of 45 cholangiocarcinoma patients (45 cancer samples) from the TCGA cohort and 30 patients (30 cancer samples and 27 nontumorous samples) from the GSE107943 cohort (See Table 1). These samples have complete clinical and transcriptomic data. Results showed that 59 genes were associated with inflammatory responses expressed in tumor and nontumorous tissues (Figure 1(a)). In a univariate Cox analysis, inflammation response-related genes were found to be linked to OS (Figure 1(b)). Among the analyzed genes, 5 overlapping inflammatory response-related genes were selected for further analysis (Figures 1(c) and 1(d)).

### 3.2. Construction of a Prognostic Model

**Figure 2:**
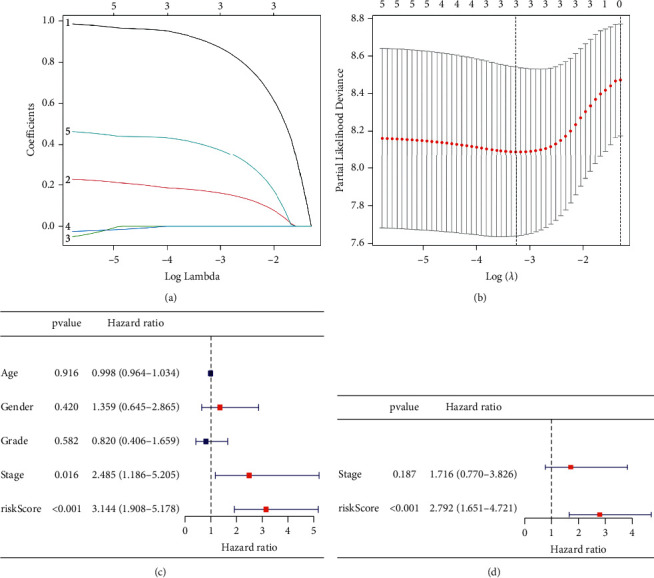
A gene signature that is associated with inflammation was generated. (a) LASSO-Cox regression analysis of possible inflammatory response-related genes. (b) The optimal value of the LASSO-Cox regression analysis. (c) Prognosis-related factors were screened using univariate Cox regression analysis. (d) Prognosis-related factors were screened using multivariate Cox regression analysis.

**Figure 3:**
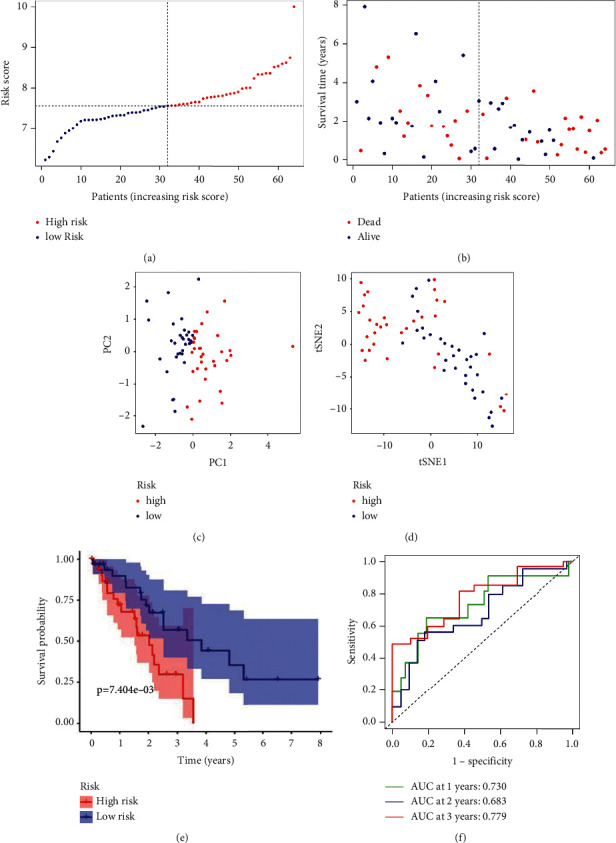
The prognostic analysis of the 3-gene signature model. (a) The median value and distribution of risk scores. (b) The prognosis status distribution. (c) PCA plot (Plotted Correlation Analysis). (d) Analysis using the t-SNE method. (e) The Kaplan–Meier curves for overall survival in high-risk and low-risk groups. (f) Overall survival AUC time-dependent ROC curves.

The expression profiles of the above 5 genes were assessed using LASSO-Cox regression analysis and a prognostic model was built (Figure 2(a)). A three-gene marker was created using the best value of *λ* (Figure 2(b)). Score = 0.899*∗*BEST1 expression level +  0.169*∗*CCL2 expression level + 0.395*∗*PLAUR expression level. Patients were divided into low-risk and high-risk groups based on the median cut-off value (Figure 3(a)). Confounding factors such as age, gender, and tumor stage were evenly distributed between low-risk and high-risk groups (Table 2). According to the scatter chart, individuals at high-risk are more likely to die from cancer than those at low-risk (Figure 3(b)). According to PCA and t-SNE analysis, individuals in different risk categories were scattered in two directions (Figures 3(c) and 3(d)). Patients at high-risk had a significantly shorter OS than those at low-risk, according to the Kaplan–Meier curve (Figure 3(e), P0.001). Time-dependent ROC curves were produced to investigate survival prediction using the prognostic model, with the area under the curve (AUC) reaching 0.730 at 1 year, 0.683 at 2 years, and 0.779 at 3 years (Figure 3(f)).

### 3.3. Independent Prognostic Value of the 3-Gene Signature and Association with Clinical Features

**Figure 4:**
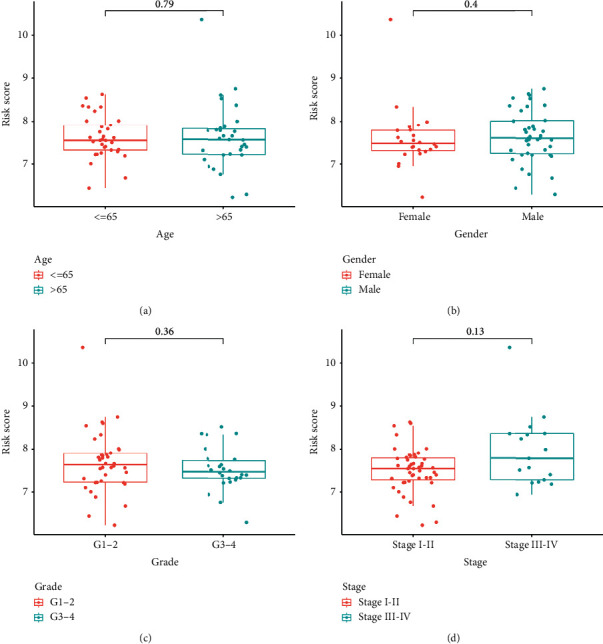
(a) Age, (b) gender, (c) tumor grade, and (d) tumor stage were used to split the risk score into various groups.

We conducted both univariate and multivariate Cox analyses of covariates to evaluate if the risk score was an independent predictor of OS. In a univariate Cox analysis, the risk score in the total population was significantly related to OS (HR = 3.144, 95% CI = 1.908–5.178, *P* < 0.001) (Figure 2(c)). Multivariate Cox analysis demonstrated that the risk score remained an independent predictor of OS after controlling for additional confounding variables (HR = 2.792, 95% CI = 1.651–4.721, *P* < 0.001) (Figure 2(d)). No meaningful association between the risk rating and clinical characteristics of cholangiocarcinoma patients, including age, gender, tumor grade, and stage, was found (Figures 4(a) and 4(d)).

### 3.4. Immune Status and Tumor Microenvironment Analysis

**Figure 5:**
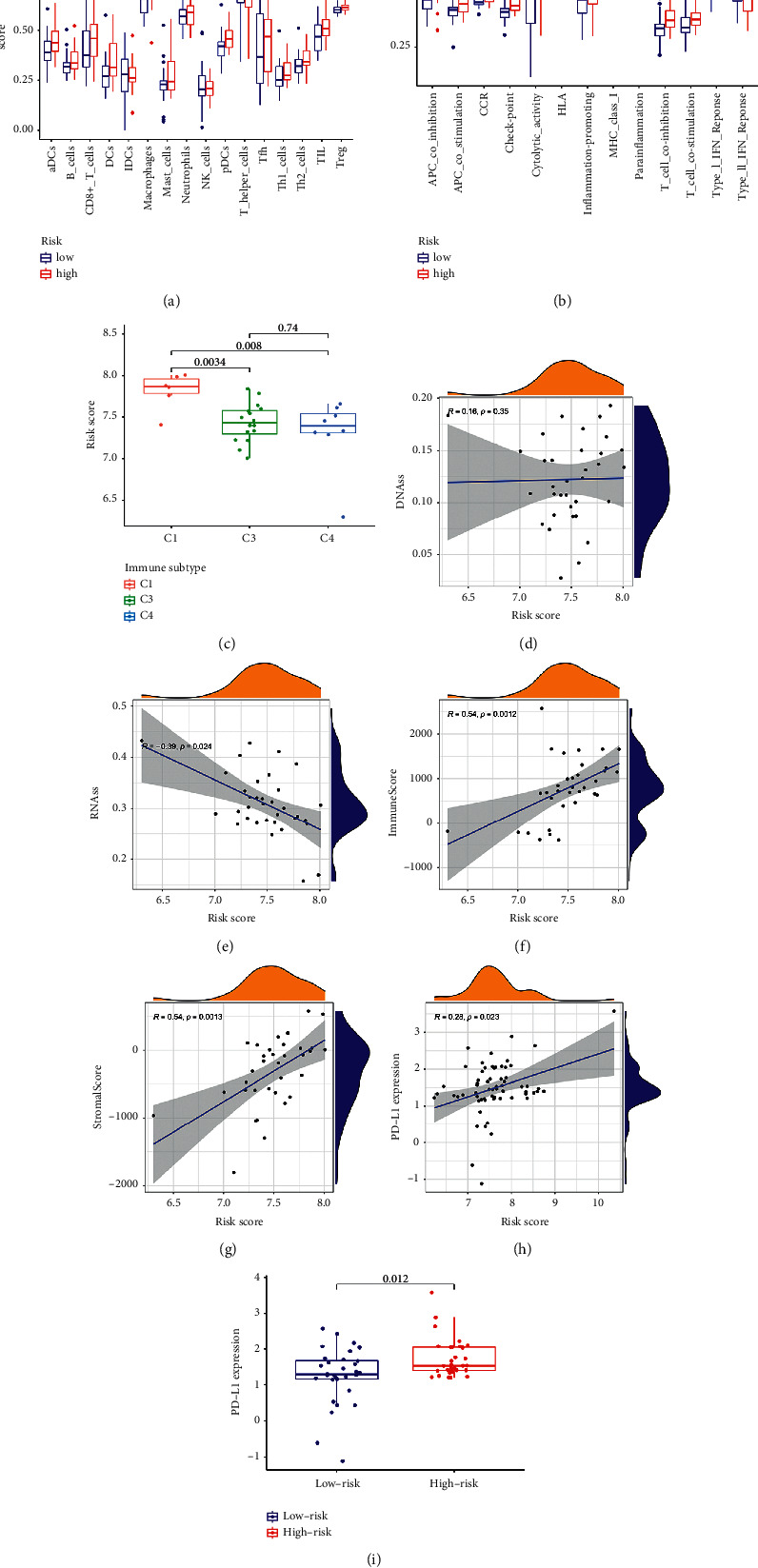
(a) The immunological status of different risk groups and the association between risk score and surrounding tissue. (b) The ratings of 16 immune cells and 13 immune-related functions were displayed using boxplots. (c) An assessment of prediction models for different subtypes of immune infiltration. Correlation among PD-L1 expression and RNAss, DNAss, stromal score, immunologic scoring system, and risk score. (d–i) The levels of PD-L1 expression in various risk groups were compared.

To study the relationship between risk score and immunological condition, ssGSEA was used to calculate the enriched scores of various immune cell subpopulations, associated components, and pathways. In the high-risk group, the antigen presentation pathways, including aDCs, pDCs, APC co-inhibition, APC co-stimulation, HLA, and MHC class I, were significantly higher (all adjusted *P* < 0.05, Figures 5(a) and 5(b)). Furthermore, the high-risk group had larger proportions of Th1 cells, Th2 cells, TIL cells, Treg cells, T cell co-stimulation, and T cell co-inhibition compared to the low-risk group, indicating differences in T cell regulation between the two groups.

To investigate how the risk score was linked to immunological components, the association between the risk score and immune infiltrates was studied. C1 (wound healing), C2 (INF-g dominating), C3 (inflammatory), C4 (lymphocyte deficient), C5 (immunologically silent), and C6 (tumor-suppressing) immune infiltrates were observed in human malignancies, ranging from tumor-promoting to tumor-suppressing (TGF-b dominant). The C5 and C6 immune subtypes were eliminated from the study because no patient specimens in HCC belonged to the C5 immune subtype and that merely one sample belonged to the C6 immunological subtype. The correlation between the immune infiltrate of cholangiocarcinoma in the cohort and the risk score was investigated. Results showed that high-risk scores highly associated with C1, while low-risk scores were strongly correlated with C3 and C4 (Figure 5(c)).

The RNA stemness score (RNAss) and DNA stemness score (DNAss) based on DNA methylation patterns were used to ascertain cancer stemness. Stromal and immune scores were used to estimate the tumor immune microenvironment. The correlation study was performed to investigate whether the risk score was linked to cancer stem cells and the immune microenvironment. Results revealed that the risk score was highly associated with RNAss rather than DNAss, and it was positively correlated with both immunologic and stromal ratings (Figure 5(d)–5(g)). The PD-1/PD-L1 pathway plays a crucial role in the immune evasion of cancer. The degree of expression of the immunological checkpoint PD-L1 was a vital indicator for specific targeting. The expression level of PD-L1 was considerably higher in the highest quintile than in the lowest quartile (Figure 5(i)), and immune checkpoint expression levels were positively correlated with risk assessment (Figure 5(h)).

### 3.5. Prognostic Gene Expression and Cancer Cell Sensitivity to Chemotherapy

**Figure 6:**
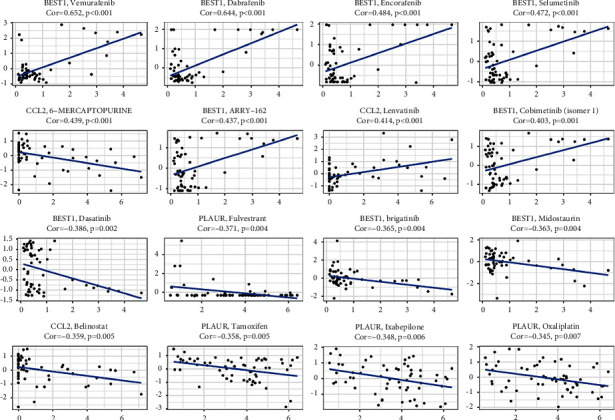
The relationship between prognostic gene expression and medication sensitivity as a scatter plot.

The expressions of prognostic genes in NCI-60 cell lines were compared to drug sensitivity and it was found that all prognostic alleles were linked to chemotherapeutic treatment sensitivity (*P* < 0.01) (Figure 6).

## 4. Discussion

In this study, we investigated the expression of 200 inflammatory response-related genes in cholangiocarcinoma tissues and analyzed their correlation to the prognosis. 49 DEGs were eliminated from the TCGA and GEO cohorts. In a univariate Cox analysis, five of the DEGs were associated with overall survival. Three inflammatory response-related genes were incorporated into a predictive model using LASSO regression analysis, namely Homo sapiens bestrophin 1 (BEST1), Chemokine (C–C motif) ligand 2 (CCL2), and plasminogen activator, urokinase receptor (PLAUR). Depending on their median risk score, patients were categorized into high-risk and low-risk groups. In a multivariate Cox regression analysis, the risk score was demonstrated to be an independent predictor of OS. The relationships between the risk score and immunological status and microenvironment were then investigated. Three inflammatory response-related genes were shown to have a high relationship with cancer cell susceptibility to antitumor drugs.

CCL2, also known as the inflammation-associated expression signature, was mostly derived from cancer-associated fibroblasts, which were components of the cholangiocarcinoma tumor microenvironment. [16, 17] Its risk score in the total population was significantly related to OS (HR = 3.144, 95% CI = 1.908–5.178, *P* < 0.001). Multivariate Cox analysis demonstrated that the risk score remained an independent predictor of OS after controlling for additional confounding variables (HR = 2.792, 95% CI = 1.651–4.721, *P* < 0.001). By stimulating the STAT3-CCL2 signaling pathway, the FAP induces immunosuppression by cancer-associated fibroblasts in the tumor microenvironment. The TWEAK/Fn14 signaling pathway may also increase the development and progression of cholangiocarcinoma niches through the downstream target CCL2. [18] Due to the lack of investigations on these genes, it remains unclear if BEST1 and PLAUR affect the prognosis of cholangiocarcinoma through inflammatory response and tumor microenvironment.

To gain a better knowledge of the connection between the risk score and immunological elements, a study on the role of risk rating in immune infiltration type was conducted. We observed that a higher risk score was substantially linked to C1, whereas a lower score was clearly connected to C3 and C4, meaning that C1 encourages cancer initiation and development while C3 and C4 are good preventative factors [19]. Furthermore, macrophages and regulatory T cells (Treg cells) presented more in the high-risk group than in the low-risk group. Because of their roles in the immunological invasion, scientists have associated a higher number of tumor-associated macrophages and Treg cells with a worse prognosis. When utilized as tumor immunotherapies, anti-PD-L1 antibodies, for example, have shown clinical activity in a variety of cancer types. Increased immune checkpoint suppressed antitumor immune responses from T cells by increasing the expression of PD-1 and CTLA4 receptors. In this study, immune checkpoint scores were greater in the high-risk group than in the low-risk group, and the risk score was strongly linked to PD-L1 expression. As a result, the prognostic model can forecast immune checkpoint expression levels and may be used to guide treatment decisions. Our research also has a few drawbacks. The predictive results in the study will need further supportive data from experiments, and the regulatory mechanism of inflammation response-related gene profile regulators on tumor growth as well as the immune microenvironment is unknown, necessitating additional research to gain a better understanding.

In conclusion, our research identified three genes involved in the inflammatory response as a new predictive signature. The signature was found to be associated independently with overall survival and to have played a role in functional analysis, tumor microenvironment, and treatment compassion, providing insights into cholangiocarcinoma prognosis. The mechanism underlying the association between inflammatory response-related genes with tumor immunity in cholangiocarcinoma remains unclear. Furthermore, these genes could be exploited to develop new antitumor medications as therapeutic alternatives.

## Figures and Tables

**Table 1:** The characteristics of patients from various cohorts at the start of their treatment.

Characteristics	TCGA cohort	GSE107943 cohort
	*N* = 45	*N* = 30
Age		
≤65	21 (46.7%)	16 (53.3%)
>65	24 (53.3%)	14 (46.7%)

Gender		
Female	25 (55.6%)	6 (20.0%)
Male	20 (44.4%)	24 (80.0%)

Grade		
Grades 1-2	23 (51.1%)	23 (76.7%)
Grades 3-4	22 (48.9%)	7 (23.3%)

AJCC stage		
Stages I-II	31 (68.9%)	21 (70.0%)
Stages III-IV	14 (31.1%)	9 (30.0%)

**Table 2:** Patients in various risk groups have varied characteristics.

Characteristics	Low-risk cohort	High-risk cohort
	*N* = 32	*N* = 32
Age		
≤65	17 (53.1%)	16 (50.0%)
>65	15 (46.8%)	16 (50.0%)

Gender		
Female	15 (46.8%)	9 (28.1%)
Male	17 (53.1%)	23 (71.8%)

Grade		
Grades 1-2	16 (50.0%)	22 (68.7%)
Grades 3-4	16 (50.0%)	10 (31.2%)

AJCC stage		
Stages I-II	25 (78.1%)	22 (68.7%)
Stages III-IV	7 (21.8%)	10 (31.2%)

## Data Availability

The data used to support this study are available from the corresponding author upon request.
